# Machine learning identifies the dynamics and influencing factors in an auditory category learning experiment

**DOI:** 10.1038/s41598-020-61703-x

**Published:** 2020-04-16

**Authors:** Amir Abolfazli, André Brechmann, Susann Wolff, Myra Spiliopoulou

**Affiliations:** 1 Faculty of Computer Science, Otto von Guericke University Magdeburg, Magdeburg, 39106 Germany; 2 Special Lab Non-Invasive Brain Imaging, Leibniz Institute for Neurobiology, Magdeburg, 39118 Germany

**Keywords:** Machine learning, Human behaviour

## Abstract

Human learning is one of the main topics in psychology and cognitive neuroscience. The analysis of experimental data, e.g. from category learning experiments, is a major challenge due to confounding factors related to perceptual processing, feedback value, response selection, as well as inter-individual differences in learning progress due to differing strategies or skills. We use machine learning to investigate (Q1) how participants of an auditory category-learning experiment evolve towards learning, (Q2) how participant performance saturates and (Q3) how early we can differentiate whether a participant has learned the categories or not. We found that a Gaussian Mixture Model describes well the evolution of participant performance and serves as basis for identifying influencing factors of task configuration (Q1). We found early saturation trends (Q2) and that CatBoost, an advanced classification algorithm, can separate between participants who learned the categories and those who did not, well before the end of the learning session, without much degradation of separation quality (Q3). Our results show that machine learning can model participant dynamics, identify influencing factors of task design and performance trends. This will help to improve computational models of auditory category learning and define suitable time points for interventions into learning, e.g. by tutorial systems.

## Introduction

One strategy to gain insight into how humans learn is to find out how they form categories or concepts^1^. Category formation or category learning is an elementary cognitive function that refers to the development of the ability to respond to common features of objects^2^ and to generalise across the huge variation of perceptual features of objects with similar behavioural relevance. When learners establish a category from a given set of objects without any explicit instruction, they need to test relevant features in a series of decisions, informed by feedback, and often extract explicit rules that define the target category. Such experiments are known as *rule-based category learning experiments* in which the category structures can be learned through some explicit reasoning process^3^.

Behavioural performance of subjects in such category learning tasks has been the focus of several formal models (e.g.^4–13^). However, researchers involved in the development of such computational models have recently come to the conclusion that existing models cannot fully explain humans’ rule-based category learning^14^. One reason for this is that participants may discern categories with transitions between learning states occurring at different points in time^15^, which is not compatible with the assumption of incremental learning as implemented in the computational models. Differences in the learners’ prior experience or explicit strategies applied to solving a given category learning task also contribute to variability. The task design itself may also introduce biases due to differences in stimulus properties and salience or may require motor responses for which participants have differing preferences and thus may increase the variance of resulting behavioural data.

During task design, confounding factors can of course be controlled for by counterbalancing, if known in advance. However, to capture the learning dynamics of individual participants, it is necessary to study how such potential confounding factors affect their variability.

In the present study, we analyse the behavioural data of a rule-based category learning experiment in the auditory domain^16^, in which participants had to learn the conjunction of two category-defining rules. Current state of the art is mainly based on studies using only one category-relevant rule. Since participants usually do not apply more complex rules at the initial phase of learning^17,18^, learning the conjunction of two rules by trial and error naturally adds to variability. We applied machine learning algorithms to the behavioural data to investigate the following questions:

- How do participants evolve towards learning the target concept, and how does this evolution depend on task design?
- To what extent does participant performance saturate, and how does this saturation depend on task design?
- How and how early can we differentiate between participants who did learn the concept and those who did not?

Answers to these questions will improve recent formal models of auditory category learning^11,13^ regarding potential differences in salience between sound features and preferences in motor response behaviour. Q3 is specifically motivated by the wish to optimise experiment duration, avoiding fatigue and its potential side effects on performance.

## Results

Before presenting our results, we give a short overview of the task design in the auditory category learning experiment^16^. The complete description can be found in the section **Methods**.

The auditory category learning experiment used tones differing in five dichotomous features, of which two determined the category to be learned: sound duration (short/long) and direction of pitch change (up/down). Each of the 76 participants was assigned randomly to one of the four *target configurations* (or *configurations*, for short): short/up (target configuration 1), short/down (target configuration 2), long/up (target configuration 3) and long/down (target configuration 4). Within each target configuration, each participant was assigned randomly with respect to the *response button* (left/right), i.e. the button to be pressed if the sound belonged to the target configuration (referred to also as “target button” hereafter). The experiment session consisted of 240 trials, of which 25% belonged to the target configuration and the remaining 75% were evenly distributed among the other three combinations of the features. After each trial, the participant received feedback on whether the response was correct or not. At the end of the experiment, the participants filled a questionnaire. From their answers, the experimenter could determine whether a participant has learned the target category (L) or not (notL: 14 out of 76).

### Results concerning Q1 on participants’ evolution towards category learning

At the beginning of the experiment, the participants do not know the target category. During the course of the experiment, the likelihood of making informed guesses is expected to increase. We refined Q1 on participant evolution into: (1) identifying the number of distinct states of performance through which participants go (possibly more than once) during the experiment, (2) the spread of performance states per participant over the whole set of trials, (3) the effect of the response button and (4) of the target configuration on the likelihood of observing a high performance state and (5) differences in this effect at the beginning of the experiment, in the mid of the experiment and towards the end of the experiment.

To address Q1, we specified an *indicator of evolution* and an *instrument* that captures the states across which participants evolve. We partitioned the 240 trials recorded during the experiment into 24 equi-sized blocks (*blockSize* = 10 trials) and used as indicator the *block performance*: we defined two performance coefficients, *blockSensitivity* (cf. Eq. (1)) and *blockSpecificity* (cf. Eq. (2)). As instrument, we used state-space modelling with a Gaussian Mixture Model (GMM) that captures the evolution of the two performance coefficients from the first to the last block as a set of *k* components.

#### Participant performance oscillates across more than two distinct states

**Figure 1:**
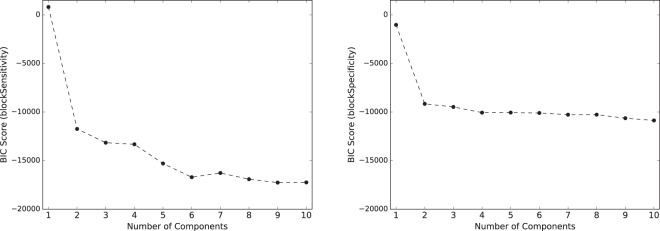
BIC score per number of components for a GMM learned on *blockSensitivity* values of all participants (left subfigure) and for a GMM learned on the corresponding *blockSpecificity* values (right subfigure), across 24 blocks of *blockSize* = 10 trials; lower BIC scores are better.

To identify the number of states of participant performance, we varied the number of components *K*, which is passed as a parameter to the Gaussian Mixture Model algorithm. For the model learned for each *K* we computed the Bayesian Information Criterion (BIC). The BIC scores for *K* = 1, 2, …, 10 are shown in Fig. 1: the left subfigure refers to GMMs on the *blockSensitivity* values of the participants, while the right subfigure refers to GMMs on their *blockSpecificity* values. Lower BIC scores are better.

As seen in both subfigures of Fig. 1, the BIC score decreases as *K* increases. This is expected, because GMMs with a large number of components *K* capture the data at a finer level. However, a large *K* also implies overfitting to the idiosyncrasies of the data. Hence, we focus on small values of *K* for which the BIC scores stagnate. For *blockSpecificity* (right subfigure), BIC decreases very slowly after *K* = 2. The BIC of blockSensitivity (left subfigure), on the other hand, still shows a pronounced drop between k = 2 and k = 3. Opting for k = 2 would furthermore produce too crude a model that distinguishes only between a state of high performance and a state of low performance, while k = 3 would also allow for a state of mid-level performance. Hence, we opt for three components and use the results of GMM with *k* = 3 in the following.

The three performance states (Gaussian components) of GMM for *blockSensitivity* had the following *θ* parameters: *θ*_*Sens*,*State*=1_ = (1.0, 0.0) – we characterise this state as *high* performance; *θ*_*Sens*,*State*=2_ = (0.6922, 0.0013); we characterise this state as *med* performance; *θ*_*Sens*,*State*=3_ = (0.358, 0.053); we characterise this state as *low* performance. The corresponding states for the performance coefficient *blockSpecificity* are: *θ*_*Spec*,*State*=1_ = (1.0, 0.0), termed *high*, *θ*_*Spec*,*State*=2_ = (0.8351, 0.0034), termed *med* and *θ*_*Spec*,*State*=3_ = (0.5916, 0.0269), termed *low*.

#### Spread of high performance blocks over the experiment’s duration

**Figure 2:**
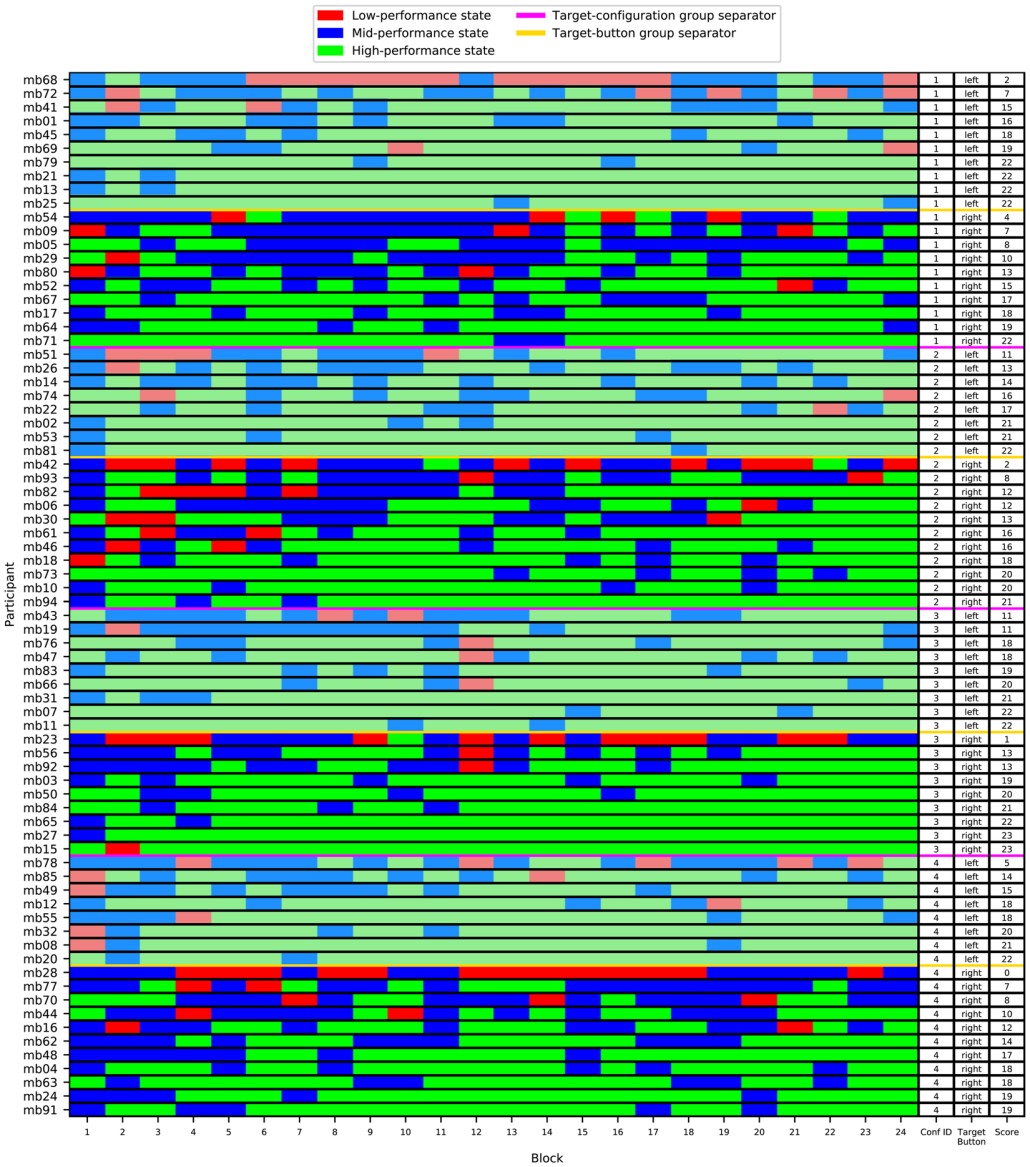
Graphical representation of each participant’s performance as a sequence of states (depicted as 24 coloured blocks of 10 trials each), where the states come from a 3-component GMM learned on *blockSensitivity*: participants are sorted based on the number of high performance blocks (green) after being grouped with respect to the configuration to which they have been exposed, and within this configuration, with respect to the response button assigned to them.

On the basis of the three performance states identified with a GMM with *K* = 3 components, we depict in Fig. 2 the sequence of *blockSensitivity* states for all participants. We show one row per participant: this row consists of 24 blocks with *blockSize* = 10 trials. The colour of a block indicates the GMM component contributing the most: a green block in Fig. 2 means that the high performance state contributes the most, i.e. that the likelihood of the *blockSensitivity* value observed for this block is maximal under the high performance state. Similarly, a blue block means that the medium performance state contributes the most, while a red block is mostly contributed to by the low performance state. In the following, we use the term *high performance block* for a block to which the high performance state contributed the most, and alike for *low performance block* and *med performance block*. (The presentation of the *blockSpecificity* states is in the **Supplementary Information**).

Fig. 2 shows the participants grouped by the configuration they have been exposed to, with purple lines separating the groups. Within each configuration, the group of participants is partitioned (by a yellow line) into two subgroups: the upper one contains the participants for whom the response (target) button was the left one (marked in pale colours), the lower subgroup contains those for whom the response button was the right one (marked in intense colours). Within each subgroup, the participants are sorted on performance, now quantified as the number of high performance blocks (green), i.e. the number of occurrences of the high-performance state. This number is shown for each participant at the rightmost column.

A cursory look at the colours shows that there are rather few low performance blocks and that the number of *adjacent* high performance blocks tends to increase when reading from left (early blocks) to the right (late blocks), towards the end of the experiment. This indicates that the participants tend to stay in the high performance state, once they have reached it. However, this tendency seems to have been influenced by the response button and by the configurations. For example, visual inspection suggests that configuration 3 seems to have led to longer sequences of adjacent high performance blocks than configuration 4.

#### Effect of the response button on the number of occurrences of the high performance state

The effect of the response button on the number of times a high performance block appears in a participant’s time series is indicated in the two rightmost columns of Fig. 2: the last column shows the number of high performance blocks per participant, while the previous column shows whether the response button for this participant was the left or the right one.

The number of participants exposed to the left vs right response button were approximately the same within each configuration and slightly different in total: 35 (response button: left) vs 41 (response button: right). We applied Mann-Whitney U tests to compare the number of high performance blocks for the two response buttons, and we found that the number of high performance blocks was significantly higher for the left button than for the right button in the analysis of *blockSensitivity* (U = 528.50, p = 0.048) and that the difference was marginally significant for the *blockSpecificity* (U = 545.00, p = 0.072). For the descriptive statistics underlying the results concerning Q1, please refer to **Supplementary Information**.

#### Effect of the target configuration on the number of occurrences of the high performance state

**Figure 3:**
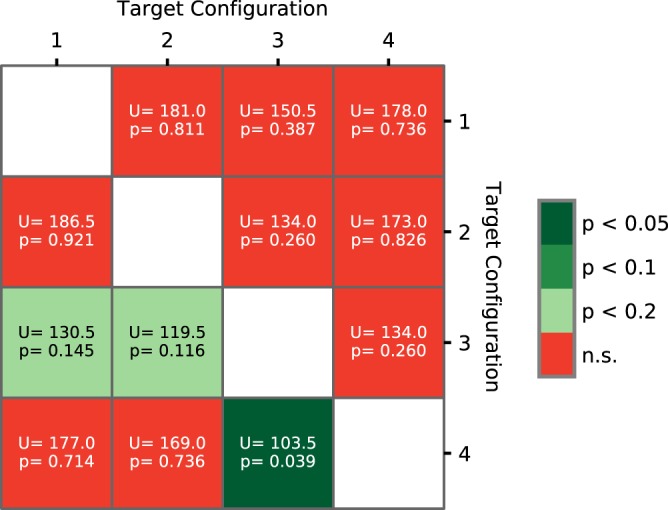
Heatmap for Mann-Whitney U tests: pairwise comparisons between any two configurations on the number of high performance blocks for *blockSensitivity* (below the diagonal) and on *blockSpecificity* (above the diagonal).

The effect of target configuration on the number of high performance blocks is shown in the heatmap in Fig. 3. The values under the diagonal show the pairwise comparisons of the target configurations for *blockSensitivity*; the values above the diagonal are for *blockSpecificity*. The colours reflect significance of the Mann-Whitney U test.

It is stressed that the purpose of this analysis (and of the next one) was not to investigate the significance of performance differences among the four configurations. Rather, we aimed to detect potentially confounding effects of the configurations, which need to be taken into account when interpreting the dynamics of participant performance. Therefore, we did not correct for multiple comparisons and we set the significance threshold at 0.2.

Fig. 3 shows that for *blockSensitivity*, configuration 3 leads to a significantly larger number of high performance blocks than configuration 4, and to marginally higher numbers than the configurations 1 and 2. The differences for *blockSpecificity* are not significant.

#### Timing of the effects of the target configuration

**Figure 4:**
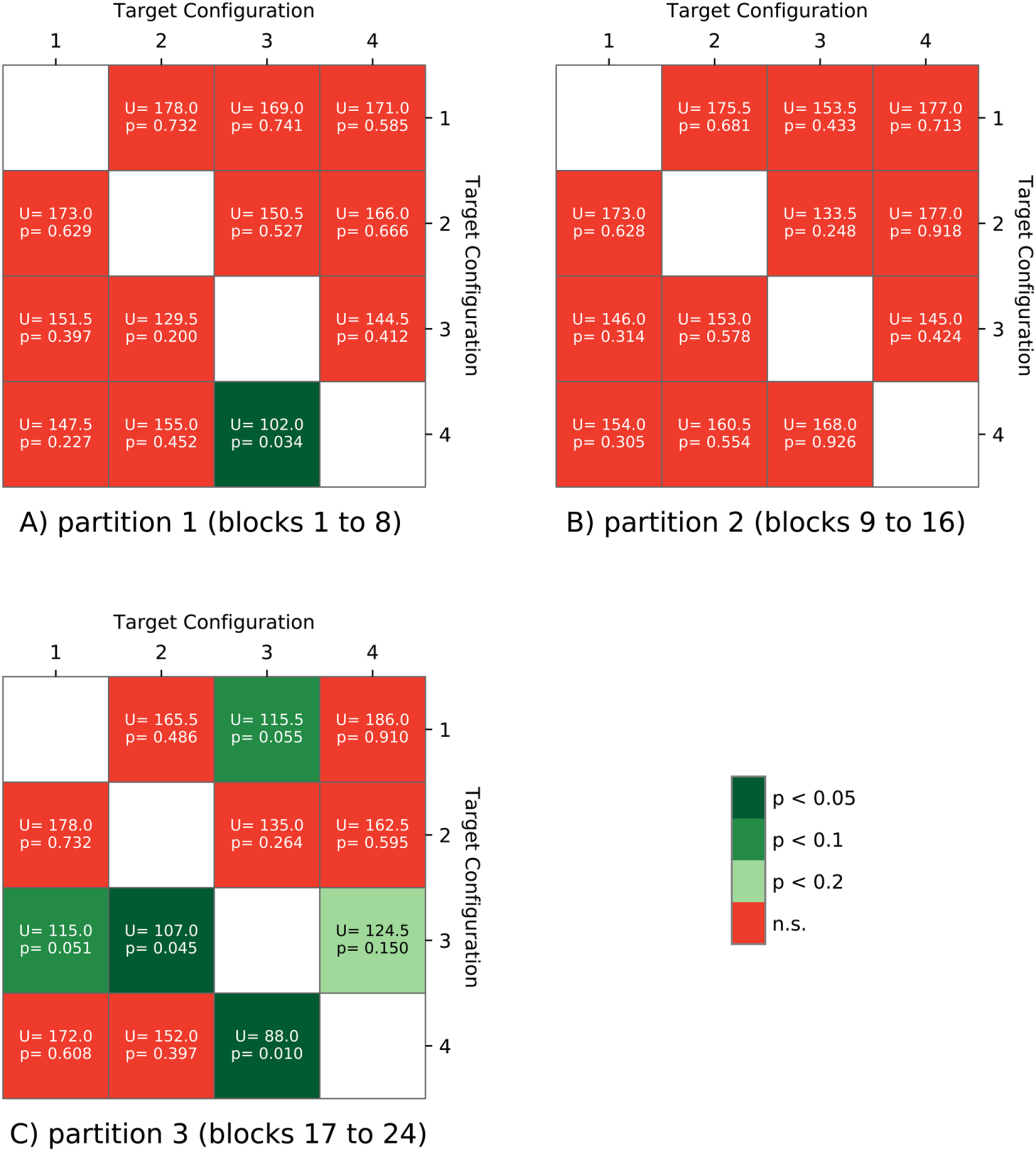
Heatmaps for Mann-Whitney U tests concerning the number of high performance blocks in each partition: pairwise comparisons between any two configurations with respect to high performance blocks inside the partition for *blockSensitivity* (below the diagonal) and for *blockSpecificity* (above the diagonal).

To investigate when the target configuration exhibits its effect on participant performance, we grouped the 24 blocks into three partitions: the first partition (partition 1) consists of the 8 blocks at the beginning of the experiment, the last partition (partition 3) consists of the 8 blocks at the experiment’s end, and the mid partition (partition 2) consists of the 8 mid-experiment blocks. The heatmaps in Fig. 4 refer again to the results of Mann-Whitney U tests: for each two configurations, we compared the number of high performance blocks for *blockSensitivity* (below the diagonal) and *blockSensitivity* (above the diagonal) in partition 1, in partition 2 and in partition 3.

Figure 4 indicates that the performance advantage of configuration 3 is traceable in the last partition and, less intensely, in the first partition. In the last partition, configuration 3 leads to a significantly higher number of high performance blocks for *blockSensitivity* than configurations 2 and 4, and a marginally significant advantage in comparison to configuration 1. The differences to configurations 1 and 4 are also marginally significant for *blockSpecificity*. In the first partition, we see only a significant difference to configuration 4 on *blockSensitivity*. In the mid partition, there are no significant differences.

These results indicate that both the configuration and the response button affect the number of occurrences of high performance blocks, and that configuration effects occur most strongly towards the end of the experiment. This serves as a warning of the confounding effects of target design on participant performance.

### Results concerning Q2 on early performance saturation

To address Q2, we specify an *indicator of saturation*, namely cumulative performance over time, again modelled across the coefficients of sensitivity and specificity, but being computed from the first trial to the trial under inspection, i.e. as *cumulativeSensitivity* (cf. Eq. (3)) and *cumulativeSpecificity* (cf. Eq. (4)). To suppress the idiosyncrasies of different participants, we aggregate into the *mean of cumulativeSensitivity* and the *mean of cumulativeSpecificity* over all participants, for each configuration and response button.

**Figure 5:**
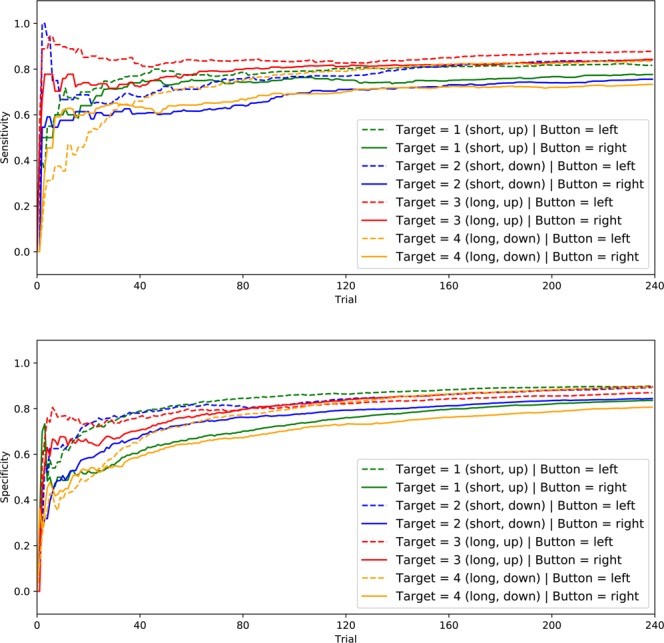
Means of *cumulativeSensitivity* (upper part) and *cumulativeSpecificity* (lower part) for each configuration and response button.

Figure 5 shows the curves of these means. We used dashed lines for the curves when the response button was the left one, and solid lines when the response button was the right one.

We focus on the curves of *mean cumulativeSensitivity* first (upper subfigure of Fig. 5). As expected, all curves start with low values in the first few trials. Then, the curves of configuration 3 (both buttons) and configuration 2 (left button only) show the fastest increase. The curves of the right button seem to saturate earlier, but reach lower values than the curves of the left button. The curves of the left button of configurations 2 and 3 exhibit a drop and a plateau, before becoming almost perpendicular to the horizontal axis. The curves of configuration 4 raise slower than those of other configurations, and the curves of the left and right button are crossed.

The curves of *mean cumulativeSpecificity* (lower subfigure of Fig. 5) show similar patterns to the curves of the other performance coefficient, except that the saturation is slower and the performance at the beginning is more homogeneous. The curves of configuration 4 cross around the 30th trial; the curves of the left button increase thereafter, both for the *mean cumulativeSpecificity* and the *mean cumulativeSensitivity* coefficient. In configuration 3, the curves also cross, albeit at a much later trial, and become almost identical thereafter.

All curves indicate that configuration and button affect the means of both cumulative performance coefficients. The configuration appears to have a stronger effect on the dynamic of performance than the button. However, for a given configuration, the means of both coefficients are higher for the left than for the right button: for configurations 1 and 2, this is the case during the whole experiment, while for configuration 4 the curves cross each other (for both coefficients). For configuration 3, the curves converge for *mean cumulativeSensitivity* and cross each other for *mean cumulativeSpecificity*.

These results indicate that the performance of the participants saturates early and that some configurations lead to higher final (saturated) performance.

### Results concerning Q3 on early separation between L and notL

We addressed Q3 by using as *instruments* (a) feature extraction upon block performance curves and (b) a binary classification model trained on two subspaces of these features as an indicator of the quality of contribution of the individual features to class separation.

Using the results of the end-of-experiment questionnaire, we modelled the separation between participants who were able to describe the rule behind the target concept (L) and those that were not (notL) as a binary classification problem. The class distribution is skewed, with the minority class notL covering 18.4% of the participants.

Our results for Q1 and Q2 indicate that performance improves very little after the first few blocks of trials. Building upon this result, we devised a set of handcrafted features that capture participant performance at the end of the experiment and at specific earlier time points, namely after each 40-trial block. This set of features is depicted in Table 1. It also contains the configuration and response button, since both were found to have an effect on participant performance. For the handcrafted numerical features in Table 1, we increased the *blockSize* to 40 trials, since the *blockSize* = 10 used for Q1 is too fine grained for a task on how early to stop the experiment. It is also stressed that the performance states identified by the GMM used all data from the 1st to the last trial for learning, and thus cannot be used to decide whether the experiment can be stopped before the last trial.

**Table 1:** Handcrafted set of features for classification of participants into the two classes with labels L/notL (cf. last row): the features capture participant performance as *blockSensitivity* and *blockSpecificity*, using larger blocks of *blockSize* = 40; response button and target configuration are also considered as features.

Variable name	Descrption	Type	Possible values
sens_1_to_40	*blockSensitivity* of block 1 (1st to 40th trial)	numerical	[0, 1]
sens_41_to_80	*blockSensitivity* of block 2 (41st to 80th trial)	numerical	[0, 1]
sens_81_to_120	*blockSensitivity* of block 3 (81st to 120th trial)	numerical	[0, 1]
sens_121_to_160	*blockSensitivity* of block 4 (121st to 160th trial)	numerical	[0, 1]
sens_161_to_200	*blockSensitivity* of block 5 (161st to 200th trial)	numerical	[0, 1]
sens_201_to_240	*blockSensitivity* of block 6 (201st to the last trial)	numerical	[0, 1]
spec_1_to_40	*blockSpecificity* of block 1 (1st to 40th trial)	numerical	[0, 1]
spec_41_to_80	*blockSpecificity* of block 2 (41st to 80th trial)	numerical	[0, 1]
spec_81_to_120	*blockSpecificity* of block 3 (81st to 120th trial)	numerical	[0, 1]
spec_121_to_160	*blockSpecificity* of block 4 (121st to 160th trial)	numerical	[0, 1]
spec_161_to_200	*blockSpecificity* of block 5 (161st to 200th trial)	numerical	[0, 1]
spec_201_to_240	*blockSpecificity* of block 6 (201st to the last trial)	numerical	[0, 1]
TargetConf	(target) configuration: determines the target category	categorical	#1, #2, #3, #4
Button	response button	categorical	left, right
Learner	acquired from the end-of-experiment questionnaire	binary	notL:0, L:1

We organised these features into five subspaces, i.e. subsets of features: Subspace_upto_40 contains all the performance coefficients of the participants in block 1, i.e. sens_1_to_40, spec_1_to_40 (cf. Table 1); Subspace_upto_80 contains all features of Subspace_upto_40, as well as the performance coefficients of the participants in block 2; …; Subspace_upto_200 contains all the performance coefficients of the participants in blocks 1 to 5. All subspaces also contain the features describing the response button and the target configuration.

For class separation, we trained CatBoost on the complete feature space and each of the five feature subspaces and compared classification performance and top-ranked features. As performance measure we used balanced accuracy, since the class distribution is skewed. For variable ranking, we used an inherent scoring mechanism provided by CatBoost itself.

The balanced accuracy values after stratified 5-fold cross validation are shown in Table 2.

**Table 2:** Balanced accuracy of CatBoost over different feature subspaces vs. complete feature space. Performance values above our criterion of 90% are highlighted in bold (values in parentheses show 95% confidence intervals).

Subspace_upto_40	Subspace_upto_80	Subspace_upto_120	Subspace_upto_160	Subspace_upto_200	Complete feature space
86.6% (±17.2%)	86.8% (±17.1%)	86.2% (±17.5%)	88.8% (±16%)	**92.8% **(±13.1%)	**97.7%** (±7.6%)

Table 2 shows that CatBoost can separate very well between L and notL. Obviously, the balanced accuracy is maximal (97.7%) when the performance of all trials is taken into account. Still, the performance values at the early blocks (cf. first three subspaces) already allow for a good class separation; this agrees with the early trends identified on cumulative performance. Subspace_upto_200, which considers the performance up to and including the 200th trial is above 90%. Hence, performance measures taken 40 trials before the end of the experiment are indicatory of whether a participant will learn the category or not.

**Figure 6:**
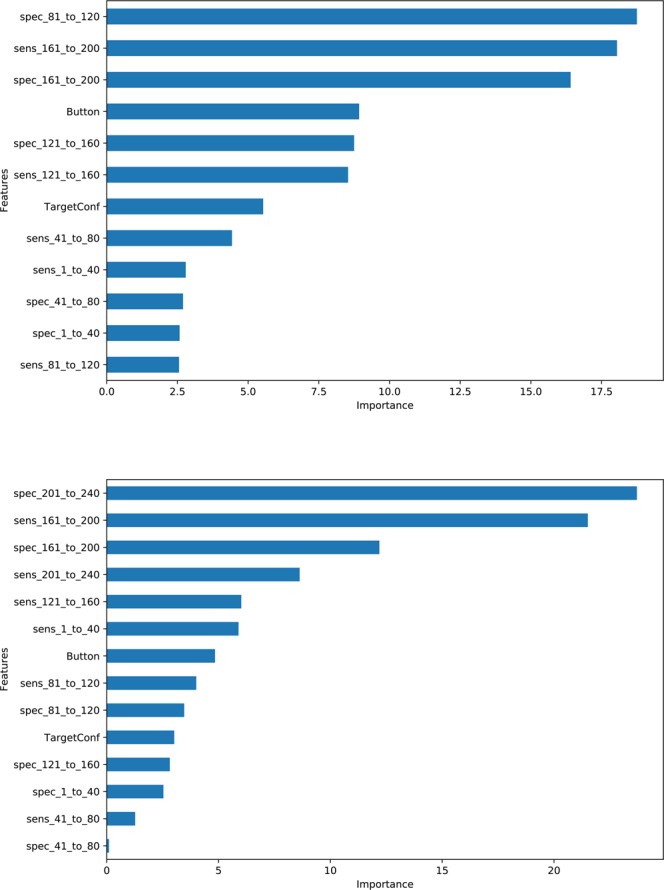
CatBoost feature ranking - upper part: feature subspace upto_200, balanced accuracy = 92.8% - lower part: the whole feature space, balanced accuracy = 97.7%.

To identify the features that contribute the most to class separation, we show in Fig. 6 the results of the internal feature ranking of CatBoost on feature importance. The upper subfigure of Fig. 6 refers to Subspace_upto_200, while the lower subfigure refers to the whole feature space. The two subfigures agree that features ‘sens_161_to_200’ and ‘spec_161_to_200’ are among the top-3 in both Subspace_upto_200 and in the complete feature space.

Hence, the performance of the participants between the 161st and the 200th trial strongly contributes to the separation between L and notL. Moreover, the separation quality (as balanced accuracy) is close to the very high value achieved when considering all trials. This indicates that the experiment can be concluded earlier – the last 40 trial block could be skipped.

## Discussion

The Gaussian Mixture Model of participant evolution towards learning revealed three states of performance, distributed unevenly across time. We found that the trend towards stable high performance is affected both by the response button (left or right) and by the target sound configuration. This agrees with the conclusions from visual category learning that “methodological details of learning experiments can have important implications” and that “sometimes seemingly inconsequential methodological details can prove critical”^19^. For example, the number of high performance blocks was higher among participants who had to press the left button with their index finger when indicating a target as opposed to those who had to press the right button with their middle finger. This is an unexpected finding since the literature only provides sparse evidence for finger preferences. We found one early study reporting an opposing preference, namely slower reaction times when choices had to be indicated by the right index finger as compared to the right middle finger^20^. Our result on the preference for the index finger when indicating a choice may be explained by the habit of using a computer mouse that developed since the 80ies. However, we are not aware of any similar finding, e.g. from usability research.

Besides this motor preference, we observed a perceptual preference. The number of high performance blocks was larger when the target category was long/up (configuration 3) as compared to the other three target sound configurations. This finding can possibly be linked to an inherent meaning of sounds with rising pitch as warning cues^21^ or the usage in language intonation as prosodic indicator of a question which, other than a statement, requires an action in form of an answer. This effect is strongest in the last partition of the experiment (cf. Fig. 4, partition C), where the differences between the configuration 3 and the other configurations are significant for the high performance blocks. We can only speculate why the superiority of configuration 3 was not evident in the mid partition of the experiment (cf. Fig. 4, partition B). We suggest that the habit of responding to sounds with rising pitch has a stronger impact on phases of the experiment where the participant either needs to guess (initial phase) or when learning has saturated (last partition). Finally, the analysis of the cumulative trends showed a combined effect of target sound category and response button due to an initial preference to press the left button for long, upward sounds and to press the right button for long, downward sounds.

In conclusion, the approach of differentiating states of high, medium and low performance by applying GMM shed light on the complex interplay between target configuration and the impact of the response button. These results contribute important information for improving formal models of category learning in the auditory domain. For example, the preferences in perceptual salience and motor response output can be considered in recurrent neural networks^13^ by changing connectivity weights in the sensory imput or motor output layers of the network whereas in cognitive modelling using ACT-R^11^, initial activation values of chunks for rule selection can be adapted accordingly. The fact that current versions of these models do not make a priori assumptions about preferences of human learners may explain their lower average performance or need for many more trials as compared to human learners. The current results now allow us to implement more realistic models of our auditory category learning paradigm. This will then provide the means to make predictions about neural correlates of dynamic decision making during the course of learning where different subjects make use of different ways of generating (initial) hypotheses about the target category by relying on prior schemata reflected in their preferences in sensory or motor processing. Moreover, computational models that include the interplay between implicit, reinforcement learning mechanisms, i.e. the neural dynamics of associative learning linking sensory cortex, basal ganglia system and the motor output system, with explicit rule discovery supported by prefrontal brain areas can be developed to meet the demand of formal explanations of the highly complex details of rule-based category learning^14^.

Such an approach of combining computational modelling with experimental data on category learning, representing a fundamental aspect of cognition, may also contribute to the unsolved question of how to correctly deliver formative feedback interventions^22,23^ to promote the learning of new concepts.

Our results show that CatBoost and feature ranking can provide information about when such interventions may take place. For the analysed experiment, the number of false alarms between trials 81 and 120 and the number of hits between trials 161 and 200 provide good indication of whether a participant will be able to explicitly report the rules for categorisation. On the basis of these results, we plan to investigate the effects of such interventions into learning by exploiting segments of the original time series for class separation.

## Methods

### Experiment on human learning

#### Participants

76 participants (38 female, 38 male, between 18 and 35 years old) performed the experiment in a sound-attenuated chamber in front of a computer. Informed consent was obtained from all participants of the study which was approved by the ethics committee of the University of Magdeburg, Germany. All research followed the tenets of the Declaration of Helsinki.

#### Stimuli and task

A set of frequency-modulated tones served as stimuli for the category learning task. The tones differed in duration (short, 400 ms, vs. long, 800 ms), direction of pitch change (up vs. down), loudness (low intensity, 76–81 dB, vs. high intensity, 86–91 dB), frequency range (five low, 500–831 Hz, vs. five high frequencies, 1630–2639 Hz) and speed of modulation (slow, 0.25 octaves/s, vs. fast, 0.5 octaves/s), resulting in 2  ×  2  ×  2  ×  2  ×  10 (160) different tones. The experiment consisted of 240 trials, each of which was characterised by the five features. The target-category was defined by the determinant features, which were the duration and the direction, resulting in one of four combinations: short/up, short/down, long/up and long/down. This corresponds to four possible *target configurations*, enumerated in the first column of Table 3: in each configuration, one of the combinations was defined as “target” category (second column); it occurred in 25% of the trials. The randomisation was such that the four different combinations of direction and duration occurred equally often in each block of 10 trials.

**Table 3:** Experiment participants, as assigned to configurations.

Conf ID	Target	Participants	
		left button (L:32, notL:3)	right button (L:30, notL:11)
1	short, up	10 (L:10, notL:0)	10 (L:9, notL:1)
2	short, down	8 (L:8, notL:0)	11 (L:6, notL:5)
3	long, up	9 (L:8, notL:1)	9 (L:6, notL:3)
4	long, down	8 (L:6, notL:2)	11 (L:9, notL:2)

The last two columns of Table 3 (numbers before the parentheses) show the number of participants allocated to each configuration of target sound and response button. For about half of the participants, the target category was associated with the left button, i.e., they had to press the left button with their index finger for indicating a target stimulus and the right button with their middle finger for indicating a non-target. For the remaining participants, the target category was associated with the right button, i.e., they had to press the right button with their middle finger for target stimuli and the left button with their index finger for non-targets. Crucially, each of the 76 participants was exposed to exactly one configuration, using either the left or the right button for the target category, and the other button to indicate a non-target tone.

After the experiment, the participants had to fill in a questionnaire in which they were asked to describe the target sounds. The answers in the questionnaire showed that 14 of the 76 participants could not unequivocally identify the configuration of the target sound. The number of participants who were able to identify the target concept and those who were not, are specified by L (Learned) and notL (Not Learned), respectively, with respect to the configuration and button, inside the parentheses of the last two columns of Table 3.

### Modelling participant performance on the time series for addressing Q1, Q2 and Q3

For question Q1, we partitioned the time series into blocks of 10 trials and quantified participant *performance* as *blockSensitivity* and *blockSpecificity*, cf. Eq. (1) and Eq. (2). For question Q2, we considered *cumulative* performance, again using sensitivity and specificity as basis, cf. Eq. (3) and Eq. (4). We partitioned the time series of trials per participant to 24 blocks of 10 trials each. Since the target concept appeared in 25% of the trials and due to pseudo-randomisation, a block of 10 trials contained two or three target signals. We defined the *non-cumulative block sensitivity* (or “block sensitivity” for short) as the proportion of the signals for which the participant had pressed the target button, to all presented target signals of that block (Eq. (1)). Similarly, we defined the *non-cumulative block specificity* (or “block specificity” for short) as the proportion of target complement signals for which the participant had pressed the not-target button, to the all target complement signals of that block. (Eq. (2)).

1 $${\rm{Block}}\ {\rm{Sensitivit}}{{\rm{y}}}_{{\rm{i}}}=\frac{| ({\rm{Signal}}={\rm{Target}})\ \& \ ({\rm{Response}}={\rm{Target}}\ {\rm{Button}}){| }_{{\rm{block}}={\rm{i}}}}{| {({\rm{Signal}}={\rm{Target}})}_{{\rm{block}}={\rm{i}}}}$$

2 $${\rm{Block}}\ {\rm{Specificit}}{{\rm{y}}}_{{\rm{i}}}=\frac{| ({\rm{Signal}}\ne {\rm{Target}})\ \& \ ({\rm{Response}}\ne {\rm{Target}}\ {\rm{Button}}){| }_{{\rm{block}}={\rm{i}}}}{| ({\rm{Signal}}\ne {\rm{Target}}){| }_{{\rm{block}}={\rm{i}}}}$$

3 $${\rm{Cumulative}}\ {\rm{Sensitivit}}{{\rm{y}}}_{{\rm{i}}}=\frac{| ({\rm{Signal}}={\rm{Target}})\ \& \ ({\rm{Response}}={\rm{Target}}\ {\rm{Button}}){| }_{{\rm{trial}}=1}^{{\rm{trial}}={\rm{i}}-1}}{| ({\rm{Signal}}={\rm{Target}}){| }_{{\rm{trial}}=1}^{{\rm{trial}}={\rm{i}}-1}}$$

4 $${\rm{Cumulative}}\ {\rm{Specificit}}{{\rm{y}}}_{{\rm{i}}}=\frac{| ({\rm{Signal}}\ne {\rm{Target}})\ \& \ ({\rm{Response}}\ne {\rm{Target}}\ {\rm{Button}}){| }_{{\rm{trial}}=1}^{{\rm{trial}}={\rm{i}}-1}}{| ({\rm{Signal}}\ne {\rm{Target}}){| }_{{\rm{trial}}=1}^{{\rm{trial}}={\rm{i}}-1}}$$

Hence, for each participant we derived one time series per performance coefficient. The number of values in this time series was determined by number of blocks of trials, which depended on the *blockSize*. For Q1, we used a *blockSize* of 10 trials. For Q3, we used fewer, larger blocks of *blockSize* = 40.

### Identifying the states of participant performance with a GMM for addressing Q1

For Q1, we applied a Gaussian Mixture Model (GMM)^24^ on the aggregated time series of *blockSensitivity*, having length of $\frac{240}{blockSize}$, and alike for *blockSpecificity*. We then used the Bayesian Information Criterion (BIC) to select the number of components, as explained hereafter.

Informally, a Gaussian mixture model (GMM) is a probabilistic generative unsupervised model, which assumes that the observations (in our study: the values of the performance coefficients of the participants) are generated from a mixture of a finite number of Gaussian distributions^25^. In other words, a GMM maps the original set of continuous observable variables to a smaller set of categorical latent variables, also known as topics, states or components.

The trained model was used for inferring the performance states from the block sensitivity/specificity values of each participant. We calculated the posterior probability of the hidden variable given the *BlockSensitivity* value of each block, in order to determine the component to which the value belongs. More precisely, for each value *x* of *BlockSensitivity* and for each component *k* = 1, …, *K*, we calculated the posterior probability *P*(*Z* = *k*∣*x*, *θ*_*k*_), where *θ*_*k*_ = (*μ*_*k*_, *σ*_*k*_) denotes the parameters of k-th component and *μ*_*k*_ and *σ*_*k*_ are the mean and standard deviation of k-th component, respectively. We then assigned *x* to the component with the highest posterior probability, i.e. $arg{\max }_{k=1,\ldots ,K}P(Z=k| x,{\theta }_{k})$. The same procedure was followed for *BlockSpecificity*.

The number of components *K* is input to the GMM. To identify the optimal number of states for the *blockSensitivity* (and alike for the *blockSpecificity*, we used the Bayesian Information Criterion (BIC)^26^, which is computed as follows:

5 $$\,{\rm{BIC}}\,={\rm{ln}}\,(n)\tau -2{\rm{ln}}\,(\widehat{\Lambda })$$

where *n* is the sample size (i.e. # participants, which is equal to 76), *τ* = 3*K* is the total number of parameters estimated by the model (mixture weight, mean and standard deviation for each component of GMM) and $\widehat{\Lambda }$ is defined as the maximised value of the likelihood function of the model. BIC is an increasing function of the error variance and an increasing function of the number of parameters estimated by the model. This implies that the unexplained variation in the dependent variable and the number of explanatory variables increase the value of BIC. Hence, lower BIC implies either fewer explanatory variables, better fit, or both^27,28^.

We used BIC as follows to specify *K*: we varied the number *K* = 1, 2, …10, compute for each *K* the value of *BIC* as in Eq. (5), plotted the values, and picked the value of *K* at which the value of BIC stopped decreasing sharply.

### Classification of Participants with Categorical Boosting for addressing Q3

#### Classifier training

For Q3, we trained a classifier that separated between participants that learned the target concept (label “L”) and those that did not (label “notL”). The classifier takes as input a set of features derived manually from the time series. For classification, we used the gradient boosting algorithm CatBoost (Categorical Boosting)^29^ and its incorporated feature importance ranker^30^.

Given a behavioural dataset of participants ${\mathscr{D}}={\{({{\bf{X}}}_{i},{Y}_{i})\}}_{i=1\ldots n}$, where ${{\bf{X}}}_{i}=({x}_{i,1},\ldots ,{x}_{i,m})$ is a vector of *m* numerical and/or categorical features, and ${Y}_{i}\in {\mathbb{R}}$ is a label value, CatBoost performs a random permutation of the dataset, and for each categorical feature of participant (TargetConf and Button), it computes average label values for the participants with the same category value placed before the given one in the permutation. With this strategy, CatBoost exploits the whole dataset for training.

CatBoost builds several decision trees, where each new tree (model) approximates the gradients of the previous one. CatBoost outperforms the existing state-of-the-art algorithms, implementing gradient boosted decision trees, such as XGBoost^31^ and LightGBM^32^, on numerous real-world problems^29^.

We used the weight scaling mechanism incorporated in CatBoost that controls the balance of positive and negative weights, useful for imbalanced classes. Positive instances (L participants) were weighted equal to $\frac{\#\ \,{\rm{negative\; instances}}\,}{\#\ \,{\rm{positive\; instances}}\,}$, which in our case was equal to $\frac{14}{62}$ = 0.225. Negative instances (notL participants) all had the weight equal to 1.

#### Classifier evaluation

Since we had a skewed class distribution, we measured classifier quality as balanced accuracy^33^, defined as arithmetic mean of sensitivity and specificity of the model in binary classification problems. In other words, the balanced accuracy measures the average accuracy on each of the classes, separately. For this calculation, the total number of positives (P) and negatives (N), and the number of true positives (TP) and true negatives (TN) are considered. In our case, the positives were all the participants of the L class and the negatives were all the participants of the notL class, in one of the 5 folds used for testing in the 5-fold cross validation. The true positives are the correctly classified participants of class L and the true negatives are the correctly classified participants of class notL. The balanced accuracy is computed as follows:

6 $$\,{\rm{Balanced}}\,\ \,{\rm{accuracy}}=\frac{{\rm{sensitivity}}+{\rm{specificity}}\,}{2}=\frac{1}{2}\frac{{\rm{TP}}}{{\rm{TP}}+{\rm{FN}}}+\frac{1}{2}\frac{{\rm{TN}}}{{\rm{TN}}+{\rm{FP}}}$$

It should be noted that here sensitivity and specificity refer to the sensitivity and specificity of the CatBoost model, not those of the participants. In our case, we calculated the balanced accuracy each time on one of the 5 folds (i.e. the one used for testing) and then reported the average balanced accuracy.

## Supplementary information

Supplementary Information.

## Data Availability

All data are available on request.
