# Supplementary material for: Machine learning identifies the dynamics and influencing factors in an auditory category learning experiment
Source: Sci Rep. 2020 Apr 16;10:6548. doi: 10.1038/s41598-020-61703-x (PMC7162940; doi:10.1038/s41598-020-61703-x)
Supplement: Supplementary file 1 — Supplementary Information. [file 41598_2020_61703_MOESM1_ESM.pdf]

Machine learning identifies the dynamics and influencing factors in an auditory category learning experiment

Amir Abolfazli[1], **André Brechmann**[2,*], **Susann Wolff**[2], **and Myra Spiliopoulou**[1]

[1]Faculty of Computer Science, Otto von Guericke University Magdeburg, Magdeburg, 39106, Germany
[2]Special Lab Non-Invasive Brain Imaging, Leibniz Institute for Neurobiology, Magdeburg, 39118, Germany
[*]Correspondence to [brechmann@lin-magdeburg.de]

Supplementary Information

Additional results for Q1

In Supplementary Figure 1 (in a separate page), we depict the performance evolution of the experiment participants across the coefficient of *blockSpecificity*. The states have been learned for a Gaussian Mixture Model with $k = 3$ components, i.e. for states of low, medium and high performance for this coefficient.

Supplementary Table 1 shows the statistics (mean, standard deviation, median and interquartile range) on the number of high performance states for the coefficient *blockSensitivity* and for the coefficient *blockSpecificity*. The high-performance state is one of the three states identified by the Gaussian Mixture Model, when setting the number of components to $k = 3$ (for each coefficient separately). We depict the statistics when the target button was specified to be the right one vs. when it was specified to be the left one. Although the number of participants exposed to the right button as target button was slightly larger, we considered the numbers comparable. For both coefficients, the mean and median were higher for the left button than for the right one.

Supplementary Table 1. Summary statistics on *blockSensitivity* and *blockSpecificity* with respect to the response button: Mean, standard deviation (SD), Median and interquartile range (IQR) for the number of high performance blocks (GMM with $k = 3$ components).

		24 blocks (*blockSize* = 10)							
		blockSensitivity				*blockSpecificity*			
target button	# participants	Mean	SD	Median	IQR	Mean	SD	Median	IQR
left	35	17.00	5.19	18.00	7.00	13.63	6.98	16.00	13.00
right	41	14.34	6.22	16.00	9.00	10.93	6.44	11.00	12.50

In Supplementary Table 2, we show the statistics for the coefficient *blockSensitivity* when splitting the 24 blocks (*blockSize* is 10 trials) into three consecutive partitions - containing the first 8 blocks, the mid 8 blocks and the last 8 blocks. The computation of the performance states is the same as before (GMM with $k = 3$), but the mean, standard deviation, median and IQR have been summarised within each of the three partitions. We depict the statistics for the four target configurations and see that configuration 3 shows consistently higher mean and median values than the other configurations in the first and last partition.

Supplementary Table 2. Summary statistics on *blockSensitivity* with respect to target configuration within each of three partitions, where *partitionSize* = 8 and *blockSize* = 10: Mean, standard deviation (SD), Median and interquartile range (IQR) for number of high performance blocks (GMM with k = 3 components).

			three partitions (*blockSize* = 10 trials, *partitionSize* = 8 consecutive blocks)											
			first partition				mid partition				last partition			
		# Participants	Mean	SD	Median	IQR	Mean	SD	Median	IQR	Mean	SD	Median	IQR
configuration	1	20	4.55	2.28	4.00	4.00	4.95	2.46	5.00	4.00	5.40	2.45	6.00	3.00
	2	19	4.16	2.31	4.00	4.00	5.37	2.16	6.00	3.00	5.89	1.82	6.00	2.00
	3	18	5.06	2.53	6.00	4.00	5.78	1.92	6.00	3.00	6.78	1.86	7.00	2.00
	4	19	3.63	1.97	4.00	3.00	5.74	2.20	6.00	4.00	5.11	2.47	6.00	3.00

Supplementary Table 3 shows the corresponding statistics for the coefficient *blockSpecificity*; again, configuration 3 shows higher values, especially in the last partition.

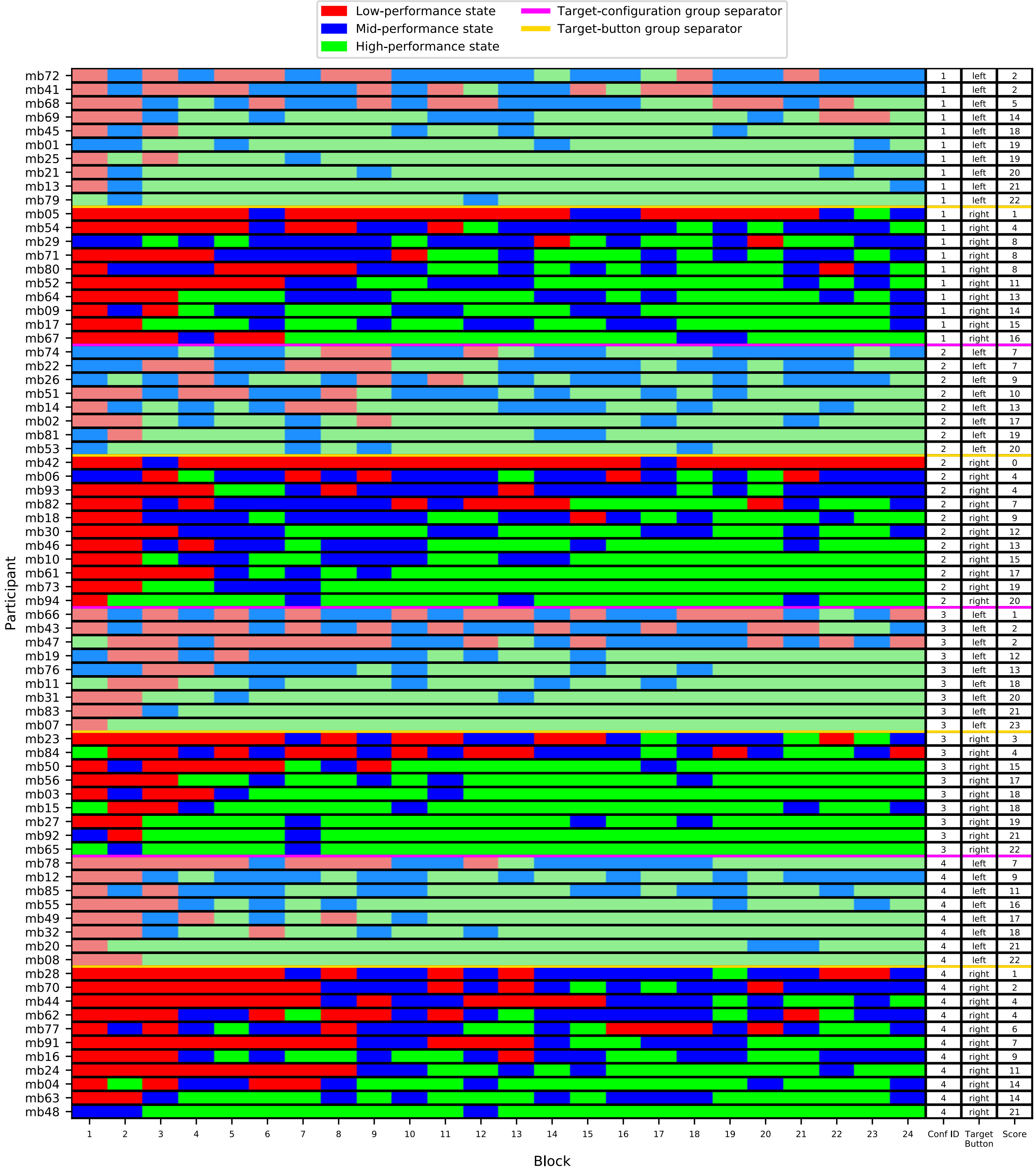

Supplementary Figure 1. Graphical representation of each participant's performance as a sequence of states (depicted as 24 coloured blocks of 10 trials each), where the states come from a 3-component GMM learned on *blockSpecificity*: participants are sorted based on the number of high performance blocks (green) after being grouped with respect to the configuration to which they have been exposed, and within this configuration, with respect to the target button assigned to them.

Supplementary Table 3. Summary statistics on *blockSpecificity* with respect to target configuration within each of three partitions, where *partitionSize* = 8 and *blockSize* = 10: Mean, standard deviation (SD), Median and interquartile range (IQR) for number of high performance blocks (GMM with k = 3 components).

			three partitions (*blockSize* = 10 trials, *partitionSize* = 8 consecutive blocks)											
			first partition				mid partition				last partition			
		# Participants	Mean	SD	Median	IQR	Mean	SD	Median	IQR	Mean	SD	Median	IQR
configuration	1	20	2.70	2.49	2.50	5.00	4.45	2.68	5.00	5.00	4.85	2.30	5.50	4.00
	2	19	2.26	1.91	2.00	2.00	4.11	2.58	4.00	5.00	5.32	2.35	6.00	3.00
	3	18	2.94	2.50	3.50	5.00	5.00	3.21	6.50	7.00	5.89	2.74	7.00	5.00
	4	19	2.16	2.26	2.00	4.00	4.16	2.83	4.00	6.00	4.95	2.39	5.00	4.00

Additional results for Q2

Supplementary Figure 2 depicts the *cumulativeSensitivity* curve (upper subfigure) and the *cumulativeSpecificity* curve (lower subfigure) for each participant, where we use colours to distinguish among the four configurations. It should be noted that despite being cumulative, these curves may move downwards. This occurs when the cumulated number of target signals perceived grows faster than the accumulated number of target signals recognised. For example, assume that a participant recognised 8 of the 10 target signals during the first 40 trials (25% of the signals are target signals), while during the next 40 trials s/he recognised only 4 of the next 10 target signals. Then, the *cumulativeSensitivity* of this participant at the 40th trial is 8/10=0.8, while at the 80th trial it is 12/20=0.6 and thus less than 0.8.

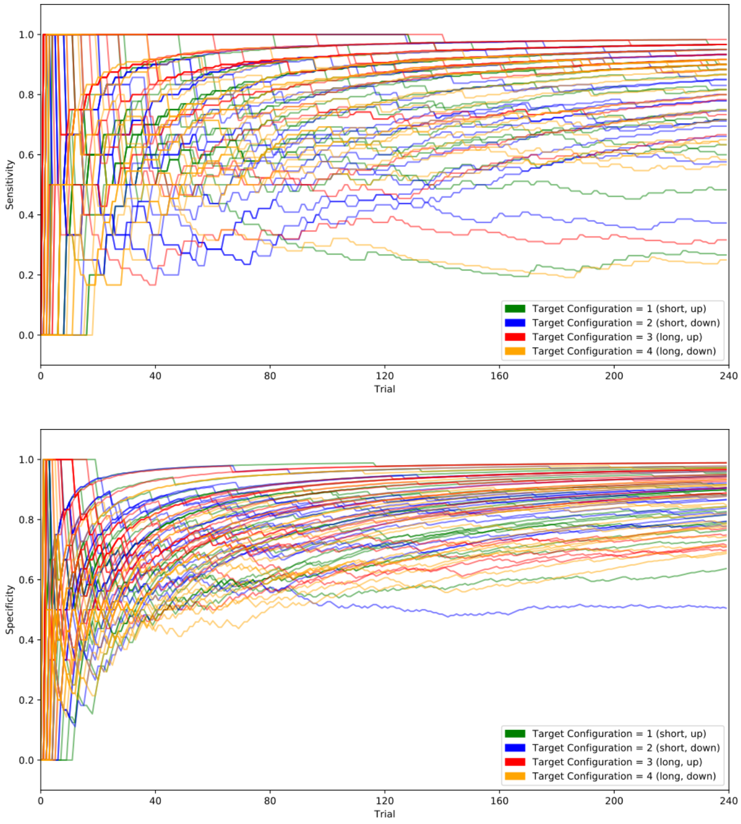

Supplementary Figure 2. Cumulative sensitivity (upper subfigure) and cumulative specificity (lower subfigure) for each participant - the curve colour indicates one of the four configurations.

Both plots of Supplementary Figure 2 show that most of the curves saturate early, reaching an almost horizontal line before the 160th trial, and sometimes much earlier. This indicates that after some time the cumulative performance does not improve any more. For *cumulativeSpecificity* (lower subfigure of Supplementary Figure 2), the increase occurs sometimes earlier than for *cumulativeSensitivity*: we see in the upper subfigure of Supplementary Figure 2 that some curves start low and remain low, while others increase slowly, after the 120th trial.
